# Changing paradigms in *Aedes* control: considering the spatial heterogeneity of dengue transmission

**DOI:** 10.26633/RPSP.2017.16

**Published:** 2017-02-08

**Authors:** Veerle Vanlerberghe, Hector Gómez-Dantés, Gonzalo Vazquez-Prokopec, Neal Alexander, Pablo Manrique-Saide, Giovanini Coelho, Maria Eugenia Toledo, Clara B. Ocampo, Patrick Van der Stuyft

**Affiliations:** 1 General Epidemiology and Disease Control Unit Institute of Tropical Medicine Antwerp Belgium General Epidemiology and Disease Control Unit, Institute of Tropical Medicine, Antwerp, Belgium.; 2 Instituto Nacional de Salud Publica CuernavacaMorelos Mexico Instituto Nacional de Salud Publica, Cuernavaca, Morelos, Mexico.; 3 Department of Environmental Sciences Emory University AtlantaGeorgia United States of America Department of Environmental Sciences, Emory University, Atlanta, Georgia, United States of America.; 4 London School of Hygiene and Tropical Medicine London United Kingdom London School of Hygiene and Tropical Medicine, London, United Kingdom.; 5 Entomological Bioassays Unit Universidad Autónoma de Yucatán, Merida Yucatán Mexico Entomological Bioassays Unit, Universidad Autónoma de Yucatán, Merida, Yucatán, Mexico.; 6 National Dengue Control Program Brazilian Ministry of Health Brasília Brazil National Dengue Control Program, Brazilian Ministry of Health, Brasília, Brazil.; 7 Department of Epidemiology Institute of Tropical Medicine “Pedro Kourí,” Havana Cuba Department of Epidemiology, Institute of Tropical Medicine “Pedro Kourí,” Havana, Cuba.; 8 International Training and Medical Research Center Cali Colombia International Training and Medical Research Center, Cali, Colombia.; 9 Department of Public Health Ghent University Ghent Belgium Department of Public Health, Ghent University, Ghent, Belgium.

**Keywords:** Dengue, epidemiology, *Aedes*, vector control, Belgium, Brazil, Colombia, Cuba, Mexico, Peru, Caribbean region, Latin America, Dengue, epidemiología, *Aedes*, control de vectores, Bélgica, Brasil, Colombia, Cuba, México, Perú, Región del Caribe, América Latina

## Abstract

Current dengue vector control strategies, focusing on reactive implementation of insecticide-based interventions in response to clinically apparent disease manifestations, tend to be inefficient, short-lived, and unsustainable within the worldwide epidemiological scenario of virus epidemic recrudescence. As a result of a series of expert meetings and deliberations, a paradigm shift is occurring and a new strategy, using risk stratification at the city level in order to concentrate proactive, sustained efforts in areas at high risk for transmission, has emerged. In this article, the authors 1) outline this targeted, proactive intervention strategy, within the context of dengue epidemiology, the dynamics of its transmission, and current Aedes control strategies, and 2) provide support from published literature for the need to empirically test its impact on dengue transmission as well as on the size of disease outbreaks. As chikungunya and Zika viruses continue to expand their range, the need for a science-based, proactive approach for control of urban Aedes spp. mosquitoes will become a central focus of integrated disease management planning.

## RESUMEN

Las estrategias actuales de control de vectores del dengue, centradas en la ejecución reactiva de intervenciones con insecticidas en respuesta a la aparición de cuadros clínicos evidentes de la enfermedad, suelen ser ineficientes, de duración limitada e insostenibles en el contexto epidemiológico mundial, caracterizado por la recrudescencia de las epidemias virales. Como resultado de una serie de reuniones y deliberaciones entre expertos, está en proceso un cambio de paradigma y ha surgido una nueva estrategia, que consiste en estratificar el riesgo de cada ciudad para concentrar y mantener los esfuerzos proactivos donde hay un alto riesgo de transmisión. En este artículo, los autores 1) describen esta estrategia de intervención específica y proactiva dentro del contexto de las características epidemiológicas del dengue, la dinámica de su transmisión y las estrategias actuales de control de *Aedes* y 2) fundamentan con fuentes bibliográficas la necesidad de demostrar empíricamente las repercusiones de esta estrategia sobre la transmisión del dengue y el tamaño de los brotes. Dado que los virus del chikunguña y el Zika siguen ampliando su alcance, uno de los objetivos primordiales de la planificación de la atención integrada de estas enfermedades estará determinado por la necesidad de adoptar un enfoque científico y proactivo del control urbano de los mosquitos del género *Aedes*.

Dengue, the most rapidly spreading vector-transmitted virus, yields a significant public health, economic, and social burden in Asia and Latin America. The importance of dengue as a public health threat is demonstrated in the exponentially growing incidence within endemic countries (1) together with a recent spreading to previously “transmission-free” areas, such as the United States (2), Croatia and France (3), and Portugal (4), following the geographic distribution of its vectors. Cases are also increasingly reported from the African continent (5). This remarkable global range expansion, reaching a global burden of 390 million infections per year, of which 96 million are symptomatic (6) and about 24 000 are fatal (7), is closely tied to global trends in population growth, unplanned urbanization, widespread travel, and globalization (8). Based on the disability-adjusted life years (DALYs) metric, dengue is responsible for an average annual loss of 658 DALYs per million population in Latin America and the Caribbean, the same order of magnitude as tuberculosis in this region (9, 10). The costs of treating clinical and severe (hospitalized) cases, the economic and social costs of loss of productive time, and the costs for controlling epidemics are exploding (9, 11).

The main mosquito vectors *Aedes aegypti* and *Ae. albopictus* can be found in the tropical and subtropical region, predominantly in urban environments, although recent empiric evidence has documented their expansion into rural areas (12–14). The four distinct but closely related dengue virus serotypes cause a wide range of disease manifestations. Dengue infection frequently remains asymptomatic but can cause classical dengue fever (an acute systemic disease characterized by fever, headache, and arthralgia/myalgia lasting for 5–7 days) or appear as a potentially lethal, severe disease with plasma leakage, with or without haemorrhagic signs. Serotype-specific immunity is lifelong but progression to more serious disease is frequently albeit not exclusively associated with secondary infection by heterologous dengue serotypes (15). Recent experimental feeding trials have documented the ability of individuals with asymptomatic infections to infect *Ae. aegypti* mosquitoes (16), leading to a new public health challenge in the surveillance and control of urban dengue.

## Limitations of current dengue control strategies

The control of dengue is mainly based on the control of its vectors at different life stages, as prevention by immunization is not yet programmatically available (but foreseeable in the future). The Phase III trials in Asia and Latin America of a leading life-attenuated candidate vaccine (yellow fever vaccine backbone) yielded efficacy rates for symptomatic dengue of 44.6% and 65.6% in people < 9 years old and ≥ 9 years old respectively (17). Unlike the vaccine for yellow fever, the development of a dengue vaccine is challenged by the need for a balanced immune response in order to address the theoretical risk of immune enhancement (18). Other vaccines are in the pipeline, such as the life-attenuated candidate that uses a DENV4 backbone, which has shown promising results in a Phase II study (18, 19). There is a growing consensus that eliminating dengue as a public health burden can only be achieved by integrating effective vector control with selective vaccination (once the vaccine is available), given the complementarity of the two approaches, and the expectation of a synergistic effect (20). The recent spread of other arboviruses transmitted by *Aedes*, such as chikungunya and Zika (21, 22), for which no vaccine is available, underscores the importance of controlling this vector within the umbrella of an integrated disease management plan.

Common approaches to control *Aedes* (i.e., those used by the disease control programs of dengue-endemic countries (23)) are directed at the insect’s larval stages and include the application of chemical larvicides or biological tools in water storage containers and mechanical destruction of non-useful containers filled with rainwater (environmental management), accompanied, or not, by community participation and a communication for behavioral impact (COMBI) strategy. These approaches can also target the adult mosquito, using methods such as outdoor and/or (less frequently) indoor insecticide space-spraying or fogging. Indoor residual spraying, when performed properly, has shown significant impacts in preventing dengue disease when applied in a top-down, regimented way (24). Some new tools, such as insecticide-treated materials, and lethal ovitraps (a device partly submerged in a cup of water that mimics container-breeding mosquitos’ breeding habitats) have recently become available (25–28). Other strategies still under evaluation are designed to reduce the *Aedes* population through the release of genetically modified mosquitoes (the Release of Insects with Dominant Lethality (RIDL) approach) or *Wolbachia*-infected mosquitoes (20). As an alternative option for dengue control, efforts can be made to decrease human–vector contact through the application of repellents at the individual level and screening doors/windows at the household level (26).

Most of the vector control measures have shown some degree of effectiveness (29) when intensive and standardized application processes are used, high coverage is reached, and sustained uptake is secured (7). However, under routine conditions, none of these measures has shown potential to halt transmission, due to, at least in part, the above-mentioned implementation challenges, as well as the increasing levels of resistance of local vectors to both larvicides and adulticides (30–32).

Current dengue control strategies in dengue-endemic countries mainly comprise dispersed and irregular control actions carried out in response to detected clinical cases, and/or massive insecticide application designed to mitigate outbreaks. These “response measures” are usually carried out several days or weeks after infection occurred, and are directed toward case houses, which may not be the major site of infection (33). The spraying of adulticide is often implemented after the peak of the epidemic (34). In contrast, a few countries conduct proactive vector control operations on a regular basis, and at nationwide scale, but the high costs (up to US$ 24 per inhabitant per year (35)) make them difficult to sustain.

## The way forward

In order to design a more rational and appropriate approach (i.e., suitable for countries where dengue is endemic or epidemic), current control strategies need to be re-thought, taking into consideration, as a starting point, the various demographic, environmental, and social conditions that drive dengue transmission dynamics. Even if it does not immediately address the distal determinants of the problem, the new paradigm in dengue control should not be designed with the assumption that transmission intensity is the same everywhere. It should consider the spatially heterogeneous character of dengue transmission risk at the city level and hence stratify geographic areas. Whenever possible the different strata should be defined and control efforts differentiated by the risk conditions that make them more or less exposed/vulnerable to dengue transmission. The research team revisited evidence on dengue epidemiology to support this new paradigm in dengue control, keeping in mind the need for a strategy that is more effective and efficient.

In the sections below, the authors 1) outline this targeted, proactive intervention strategy, within the context of dengue epidemiology, the dynamics of its transmission, and current *Aedes* control strategies, and 2) provide support from published literature for the need to empirically test its impact on dengue transmission as well as on the size of disease outbreaks.

### Identifying areas with high risk for dengue transmission.

Transmission of infectious diseases in general and vector-borne diseases specifically is known to be highly heterogeneous in terms of space and time (36–38). Mathematical models indicate that infectious disease interventions that are targeted spatially (and/or temporally) can be more effective than those applied evenly across different locations and time periods (39). Transmission of dengue has also shown spatial and temporal heterogeneity. Spatial areas of elevated risk for dengue were identified at the sub-city level in Peru and at the district level in Vietnam (40, 41). The stability of spatial patterns of dengue incidence was highlighted in research carried out by Barrera et al. (42) in a hyperendemic city in Venezuela, where 70% of reported cases over a five-year period occurred in 35% of all neighborhoods (where 55% of the city inhabitants lived). In Cairns, a non-endemic city in Australia, an analysis of the spatio-temporal dimension of dengue virus transmission demonstrated that 18 space–time clusters involved 65% of cases, and most dengue introductions leading to epidemic propagation occurred within the coastal (and much older) neighborhoods of the city (24).

Based on the malaria hotspot definition provided by Bousema (43), areas with a high risk for dengue transmission could be defined as geographic sites where transmission intensity exceeds the average level. But unlike malaria, average transmission for dengue must be framed within temporal scales because it is dependent on the amount of time that has elapsed since the dengue or a specific serotype was introduced to the area, the level of immunity to a specific serotype within the population, and the transmission intensity within an epidemic/endemic period. Various approaches can be used to assign different levels of risk to specific geographic areas, such as neighborhoods. Two of those approaches are described below. The first approach is based on measures of disease incidence; the second approach is based on measures of vulnerability.

One advantage of the approach based on disease incidence is that data on dengue cases at the population level are routinely available. However, the incidence measure approach has several drawbacks, such as only capturing data from people who have visited public health services and for whom dengue was reported as the main diagnosis (44), and the variability of symptom presentation, which is partly dependent on previous dengue exposure. In addition, these types of data are associated with the residential address of each case, rather than the actual point of transmission, which limits estimations of true location-specific incidence, given that transmission could have occurred outside the neighborhood/city where individuals live (45). In addition, a significant proportion of dengue infections are asymptomatic, even though they can still play an important role in maintaining disease transmission (16, 46). A better measure of past transmission would be dengue serotype–specific seroprevalence, which includes asymptomatic infections. Unfortunately, such data are rarely available at the population level due to the high cost of the diagnostic procedures (47). However, incidence and/or seroprevalence information can be modeled afterward, looking at space–time correlations and taking into account possible covariates.

The second approach, based on measures of vulnerability, is designed to capture multiple dimensions of risk for dengue transmission, beyond the limited predictive ability for actual transmission of one determinant used in isolation. Therefore, models based on this approach incorporate variables characterizing dengue transmission risk: entomologic (48), epidemiologic (49), demographic (50), behavioral (33), and environmental (51, 52) factors. This multifactorial approach is in line with the Mesoamerican plan for integrated dengue prevention and control (53). In a multidisciplinary meeting with dengue experts from academia and national control program managers (Havana, 2014), specific factors within these determinant groups were identified at municipality or city (the level at which control programs are organized). Epidemiologic risk per neighborhood can be characterized based on several indicators: cumulative incidence of dengue cases over a 5–10 year period, the neighborhoods where the first cases usually appear, the persistence of case incidence in/between epidemics, and the serotype(s) that are circulating. The entomologic risk per neighborhood can be explained by two main indicators: the persistence of high indices over time, and cumulative infestation levels (over a period of 2–5 years). These indicators will need to be adapted for the local context of each country, as available entomologic information differs across countries in terms of the methods used, the frequency, and the geographic coverage of surveys. As detailed information on entomologic infestation levels is often lacking or incomplete, the model should also include environmental factors that enhance the probability of vector presence. Environmental and behavioral characteristics could include local risk behaviors or risk spots for *Aedes* infestation (e.g., cemeteries, tire storage areas). For demographic factors, it was found that, along with population density (or, if more appropriate, urbanization characteristics from satellite imagery), it was important to take into account intensive human movement (54, 55). Factors such as temperature, rainfall, and altitude were not retained, as they hardly vary at the municipal or city level. However, in cases where they are deemed important, they could be added to the list of variables.

### Criteria that must be defined and questions that must be answered before adapting the control paradigm to a targeted intervention.

Before designing an intervention strategy, based on the identification of high-risk areas, certain criteria must be defined and certain research questions answered. One major criterion to define is the geographic scale at which the disease transmission areas need to be defined. To date, appraisal of heterogeneity has mainly been used operationally to identify vast subnational geographic entities (e.g., provinces) to direct dengue control actions (56). However, some researchers have observed that heterogeneity of transmission is better exploited on a smaller scale, because vector infestation levels and risk for dengue transmission can differ markedly from one neighborhood to another within the same city (57). For example, in Peru, it has been shown that neighborhoods (aggregated spatial units) have a higher level of stability as transmission-risk units than individual households (58). Based on these findings, and on the operationalization of routine programs, neighborhoods could be a better target for focusing control efforts than the household or the entire city.

A second criterion that needs to be defined/verified is the stability/persistence of dengue transmission risk within the selected geographic units. The evidence on geographic consistency of clinical dengue incidence and *Aedes* infestation is scarce, uses different geographic scales, and is mainly based on retrospective data analysis. Study results demonstrate highly variable findings on the stability of risk (40–42; 58–61). The best way to validate the spatial consistency of transmission risk in specified operational settings is to use prospective data for evaluating the consistency of an analysis in real time, or current data for evaluating the consistency of an analysis over a retrospective period.

Finally, the following question should be posed: “Do areas with elevated risk for transmission seed/fuel transmission outside these areas?” Albeit not absolutely necessary, intervention impact would increase considerably if dengue control interventions in high-risk areas led to decreased dengue transmission in the surrounding areas. Therefore, the level of control achieved should be evaluated (as “cases averted” or “reduction of transmission”), along with the benefits of the cost-effectiveness of the “targeting” interventions (described below) compared to the more expensive blanket control measures.

## Dentarget research network

DENTARGET is a multidisciplinary research network that brings together decision-makers and academics with extensive experience in dengue from five Latin American countries (Brazil, Colombia, Cuba, Mexico, and Peru) and the Institute of Tropical Medicine (Antwerp, Belgium) to develop effective and efficient novel dengue control strategies. Network members^10^ have elaborated a research plan consisting of three steps. First, they plan an investigation to identify and validate areas at high risk of dengue transmission in several municipalities per country. Once the group is confident the most appropriate neighborhoods have been identified, it designs a “targeting” intervention (proactive vector control activities in high-risk neighborhoods) to complement routine dengue control activities. The effectiveness of the intervention is assessed in terms of 1) dengue transmission (which could be expressed as averted cases), and 2) the ratio of incremental cost-effectiveness (i.e., from the perspective of the vector control program) of the “targeting” strategy versus the “routine” strategy. In the final step, the group assesses the scope and barriers for possible scaling up of the “targeting” intervention. Given the recent expansion of chikungunya and Zika viruses in the Americas, this approach will also allow for testing of the hypothesis that proactively targeting dengue hotspots could limit the transmission of other *Ae. aegypti*–transmitted arboviruses with similar eco-epidemiologic determinants. Studies in Acapulco, Mexico, found mosquito pools coinfected with both dengue and chikungunya, suggesting co-circulation of dengue (different serotypes) and chikungunya viruses within small geographic units (62). Targeting control can thus improve the cost-effectiveness of interventions in the current scenario of multiple co-circulating viruses.

## Expected breakthrough if successful

If successful, the methodologies transmission could be useful for 1) the organization of health services for case management (i.e., preparation for high-transmission season); 2) the selection of sites for sentinel surveillance (a system proposed by PAHO^11^); and 3) the development of a strategy for scaling up control efforts and/or the deployment of a future dengue vaccine. If proven effective, the targeting strategy could also benefit efforts to control the recently spreading viruses, chikungunya and Zika.

## Conclusions

An alternative dengue control strategy was identified based on the spatial heterogeneity characteristic of disease transmission. Implementation studies are under way and will provide evidence on its effectiveness and cost-effectiveness.

### Funding.

The DENTARGET network received funding from the framework agreement between the Institute of Tropical Medicine (Antwerp) and the Belgian Directorate-General for Development Co-operation. The funders had no role in content discussions or in preparation of manuscript.

### Disclaimer.

Authors hold sole responsibility for the views expressed in the manuscript, which may not necessarily reflect the opinion or policy of the *RPSP/PAJPH* or the Pan American Health Organization (PAHO).

## Footnotes

10 Rufino Cabrera Champe, Fernando Donayres, Lely Solari, Hector Gomez-Dantés, Gonzalo Vazquez-Prokopec, Pablo Manrique-Saide, Juan I. Arredondo-Jiménez, Gustavo Sánchez-Tejeda, Carlos Melo, Rodrigo Lins Frutuoso, Joscelio Aguilar Silva, Giovanini Coelho, Nildimar Honorio, Jefferson Pereira Caldas dos Santos, Bertha Nelly Restrepo, Neal Alexander, Clara Ocampo, Mabel Carabali, Alicia Reyes, Eric Martinez, Nivaldo Linares Perez, Julio Cesar Popa Rosales, Maria Eugenia Toledo, Veerle Vanlerberghe, and Patrick Van der Stuyft (http://dentarget.finlay.edu.cu/).

11 Pan American Health Organization (http://www.paho.org/pan/index.php?option=com_content&view=article&id=782:paises-americasimplementaran-un-sistema-vigilancia-integradadengue&Itemid=303).
